# Comparative Performance of Eight PCR Methods to Detect *Cryptosporidium* Species

**DOI:** 10.3390/pathogens10060647

**Published:** 2021-05-23

**Authors:** Damien Costa, Louise Soulieux, Romy Razakandrainibe, Louise Basmaciyan, Gilles Gargala, Stéphane Valot, Frédéric Dalle, Loic Favennec

**Affiliations:** 1 Department of Parasitology/Mycology, University Hospital of Rouen, 76000 Rouen, France; louise.soulieux@ecomail.fr (L.S.); gilles.gargala@chu-rouen.fr (G.G.); loic.favennec@chu-rouen.fr (L.F.); 2 EA ESCAPE 7510, University of Medicine Pharmacy Rouen, 76000 Rouen, France; romy.razakandrainibe@univ-rouen.fr; 3 CNR LE Cryptosporidiosis, Santé Publique France, 76000 Rouen, France; 4 CNR LE Cryptosporidiosis Collaborating Laboratory, Santé Publique France, 21000 Dijon, France; Louise.Basmaciyan@u-bourgogne.fr (L.B.); stephane.valot@chu-dijon.fr (S.V.); frederic.dalle@u-bourgogne.fr (F.D.); 5 Department of Parasitology/Mycology, University Hospital of Dijon, 21000 Dijon, France

**Keywords:** *Cryptosporidium*, PCR, detection, diagnosis, sensitivity, specificity

## Abstract

Diagnostic approaches based on PCR methods are increasingly used in the field of parasitology, particularly to detect *Cryptosporidium*. Consequently, many different PCR methods are available, both “in-house” and commercial methods. The aim of this study was to compare the performance of eight PCR methods, four “in-house” and four commercial methods, to detect *Cryptosporidium* species. On the same DNA extracts, performance was evaluated regarding the limit of detection for both *C. parvum* and *C. hominis* specificity and the ability to detect rare species implicated in human infection. Results showed variations in terms of performance. The best performance was observed with the FTD^®^ Stool parasites method, which detected *C. parvum* and *C. hominis* with a limit of detection of 1 and 10 oocysts/gram of stool respectively; all rare species tested were detected (*C. cuniculus, C. meleagridis, C. felis, C. chipmunk*, and *C. ubiquitum*), and no cross-reaction was observed. In addition, no cross-reactivity was observed with other enteric pathogens. However, commercial methods were unable to differentiate *Cryptosporidium* species, and generally, we recommend testing each DNA extract in at least triplicate to optimize the limit of detection.

## 1. Introduction

Human cases of cryptosporidiosis were first reported in the 1970s in children and immunosuppressed adults [1]. In 2015, the Global Enteric Multicenter Study (GEMS) described *Cryptosporidium* spp. as the second leading cause (5–15%) of moderate to severe diarrhea among infants in countries of sub-Saharan Africa and South Asia, after rotavirus [2]. At the same time, *Cryptosporidium* spp. were found to be responsible for more than 8 million cases of foodborne illnesses in 2010, and they were ranked fifth out of 24 potentially foodborne parasites in terms of importance [3,4]. In 2017 in France, the National Reference Center-Expert Laboratory (CNR-LE) for cryptosporidiosis was set up, allowing the collection and interpretation of epidemiological data thanks to the participation of members of the network. Published data from the French CNR-LE for cryptosporidiosis show that: i) even with around 250 notified cases each year, cryptosporidiosis is still largely underestimated in France, ii) cryptosporidiosis is predominant in immunocompetent individuals and especially in young children and young adults, and iii) cryptosporidiosis is over-represented in the summer [5,6]. The routine diagnosis of cryptosporidiosis still relies on light microscopy examination for many laboratories [7,8,9,10]. However, light microscopy examination lacks sensitivity, is time-consuming, and requires skilled technicians, making it an inefficient method for laboratories which are able to switch to PCR analysis [8,10,11,12]. Currently, several PCR methods are available to screen *Cryptosporidium* spp. DNA, both “in-house” and commercial methods, sometimes incorporated into multiplex panels [13,14,15,16,17]. Consequently, more and more laboratories are opting for such methods based on the practicalities of economic management. However, disparities exist in the performance of these methods. DNA extraction is essential to obtain good performance in PCR analysis and especially regarding parasitological investigations on stool samples. Some studies reported different performances of DNA extraction methods, and regarding the extraction of *Cryptosporidium* oocysts, a mechanical treatment of stool samples seems essential [18,19,20,21,22,23]. In addition to the extraction method, the removal of inhibitory substances and the gene locus targeted by related primers plays a major role in the performance of the method. One of the tasks of the CNR-LE for cryptosporidiosis is to assess the performance of available diagnostic tools. A previous work already compared performances of various extraction methods on *C. parvum* oocysts from stool samples [22]. In continuity of this work and based on the most effective extraction method, we propose a comparison of the limit of detection of eight real-time PCR methods (commercial or not) on the DNA of *Cryptosporidium* species. The main aim was to provide data to select the best methods for DNA amplification in terms of sensitivity and ability to detect human pathogenic *Cryptosporidium* species (even rare ones) in routine diagnosis.

## 2. Results

The results obtained from the four “in-house” PCR methods are summarized in Table 1. Except for the most concentrated *C. parvum* extract (10^5^ oocysts/gram), significant differences in threshold cycle (Cq) values were observed when applicable (on ANOVA test). All four “in-house” methods detected *C. parvum* DNA and *C. hominis* DNA with a limit of detection of 10^3^ oocysts/gram and 10^4^ oocysts/gram, respectively. The most sensitive “in-house” PCR method for both *C. parvum* and *C. hominis* was the method developed by the CNR-LE Cryptosporidiosis Collaborating Laboratory (University Hospital of Dijon) and described by Valeix et al. 2020 [22]. Cq values obtained with the method described by Mary et al. 2013 [15] were lower than other tested methods but analysis performed in triplicate was insufficient to detect 10 oocysts/gram for *C. parvum*, contrary to the method described by Valeix et al. PCR efficiencies were satisfactory only on *C. parvum* DNA amplification for the methods described by Fontaine et al. 2002 and Valeix et al. 2020 [13,22]. R² values were satisfactory (>0.99) only to detect *C. parvum* and for the methods described by Fontaine et al. 2002, Hadfield et al. 2011 and Mary et al. 2013 [13,14,15].

The results obtained from the four multiplex commercial methods are presented in Table 2. All four commercial methods detected *C. hominis* DNA and *C. parvum* DNA with a limit of detection of 10^3^ oocysts/gram. The best performance was obtained with the FTD Stool parasites method, with a detection of DNA corresponding to 1 oocyst/gram for *C. parvum* and 10 oocysts/gram for *C. hominis*. The Allplex GI Parasite Assay kit was the second-best method, with a detection of DNA corresponding to 100 oocysts/gram for *C. hominis* and 10 oocysts/gram for *C. parvum* but requiring a triplicate to reach the limit of detection (only 1/3 triplicates was positive to detect *C. parvum* at 10 oocysts/gram). PCR efficiencies and R² values varied greatly depending on the studied method but overall were unsatisfactory (PCR efficiency < 90% or > 110% and R² value < 0.99)

The ability to detect rare species implicated in human pathologies for each tested method is summarized in Table 3. All tested methods were able to detect the species *C. cuniculus*, *C. meleagridis*, *C. felis*, *C. chipmunk*, and *C. ubiquitum*, except the methods described by Mary et al. 2013 and Fontaine et al. 2002 [13,15]. Specificity tests performed in triplicate per condition, as described in the Methods section, revealed cross-reactivity only for the method described by Hadfield et al. 2011 [14] with *Encephalitozoon intestinalis* DNA.

## 3. Discussion

This study was designed to address questions regularly raised within the framework of scientific exchanges of the CNR-LE for cryptosporidiosis. It compared the performance of eight PCR methods to detect *Cryptosporidium* species (even rare) implicated in human infection, and their limit of detection. At first, the subject appeared to be well-investigated within the scientific community. However, in most cases, PCR performances to detect *Cryptosporidium* DNA were evaluated in cohorts from microscopically positive stool samples (probably relatively highly concentrated in oocysts), or not specifically through multiplex panels and from various extraction methods, or sometimes from DNA extracts stored for a long time [24,25,26,27,28,29,30,31,32,33]. In this study, thanks to a standardized extraction procedure (selected among the best methods regarding specific *Cryptosporidium* DNA extraction from stool samples [22]), observed PCR performances were exclusively due to the DNA amplification step. The limit of each studied PCR method was determined by assessing titrations of *Cryptosporidium* oocysts in stool samples as well as testing rare species implicated in human infection. The main interest of the study was to provide data on efficient methods for the routine diagnosis of cryptosporidiosis as a complement to extraction methods already assessed [22,23].

The results obtained generally showed similar performances between commercial and “in-house” methods in terms of limit of detection, with variations between each tested kit. Regarding *C. parvum* and *C. hominis* respectively, limits of detection generally reached at least 100 and 1000 oocysts/gram regardless of the method. Nevertheless, the limit of detection appeared optimal with the FTD^®^ method considering both *C. parvum* and *C. hominis*. Variations in limits of detection may first be explained by the genes targeted by PCR methods. Three of the four “in-house” methods target the 18S rRNA gene whose expression is estimated at 5 copies/genome (20 copies/oocyst) [15]. The “in-house” method described by Fontaine et al. 2002 targets a gene whose expression is estimated at 1 copy/genome, and indeed, its observed performance in terms of limit of detection was generally poorer than that of the three methods targeting the 18S rRNA gene. Regarding commercial methods, targeted genes were only available for FTD^®^ (DNA J-like protein, number of copies per genome not known) and Amplidiag^®^ methods (COWP gene; 1 copy/genome). Of note, the observed performance of the Amplidiag^®^ method was close to that of Fontaine et al.’s “in-house” method targeting a gene also expressed in 1 copy/genome. Comparing Table 1 and Table 2, the limit of detection of the Amplidiag^®^ method appeared slightly better than that of Fontaine et al.’s “in-house” method (for both *C. parvum* and *C. hominis*) but this was only due to DNA detection in one replicate at the very end of the PCR program. It could be explained by the heterogeneous distribution of DNA in elution volume when parasite concentrations are low. To limit this bias, and to obtain optimized performance, we recommend running each DNA extract in several replicates (at least in triplicate) or until exhaustion if possible.

Regarding the results obtained to detect rare species of *Cryptosporidium* implicated in human infections, most tested methods were able to detect rare species except the “in-house” methods of Fontaine et al. 2002 and Mary et al. 2013 [13,15]. However, a limitation of this study was the use of only triplicate of each tested *Cryptosporidium* subtype due to the amount of available positive stools. The use of more numerous rare strains could potentially improve the observed results. For the method described by Fontaine et al. 2002, they highlighted the use of a specific primer-probe set supposed to be specific for a *C. parvum* genomic DNA sequence. No cross-reactivity with other *Cryptosporidium* species was expected; however, they initially reported cross-reaction with the *C. meleagridis* genotype, which was confirmed in our study [13]. For the method of Mary et al. 2013, no rare species was detected in this study. In the original article, tests on *C. felis*, *C. bovis*, *C. cuniculus*, *C. canis*, and *C. chipmunk* were evoked in the discussion. However, in reality, corresponding results were not shown [15]. Consequently, primers and probes described in the article of Mary et al. 2013 are probably very specific to *C. parvum* and *C. hominis*. Regarding specificity in this study, performances obtained were highly satisfactory for each tested condition in concordance with the literature [13,14,15,27,34]. Cross-reactivity with *Candida albicans* DNA was tested since Mary et al. 2013 reported potential cross-reactivity with the *C. albicans* 18S rRNA gene (based on an in silico approach) and primers and probes of the PCR method described by Hadfield et al. 2011 [14,15]. For the method described by Hadfield et al. 2011, no cross-reactivity was observed with *C. albicans* but cross-reactivity was observed with *E. intestinalis*.

Finally, out of a total of 784 PCRs performed, varying results were obtained from the same DNA samples. Commercial methods (especially FTD^®^ and Allplex^®^) appeared to be valuable options for large screening to detect *Cryptosporidium* species. We recommend testing each DNA extract at least in triplicate to optimize the detection of small amounts of DNA. However, if commercial methods are able to detect rare species, results are expressed exclusively as positive or negative for *Cryptosporidium* spp. DNA detection. Consequently, to discriminate species, we recommend the use of “in-house” methods, and especially the method described by Valeix et al. 2020 [22], due to the results obtained in terms of limit of detection and the ability to detect rare species. In addition, the method described by Valeix et al. 2020 appeared to be strongly replicable, since performances in terms of limit of detection were similar to those described here, even using different stool samples [22].

## 4. Materials and Methods

### 4.1. Strains

*Cryptosporidium* spp. tested strains were obtained from the French cryptosporidiosis CNR-LE stools collection. *C. parvum* IIaA15G2R1 (n = 3), *C. hominis* IbA10G2 (n = 3) gp60 subtypes, *C. cuniculus* (n = 3), *C. meleagridis* (n = 3), *C. felis* (n = 3), *C. chipmunk* (n = 3), and *C. ubiquitum* (n = 3) were tested, all previously isolated from human clinical samples. In total, per studied method, six ten-fold range points (10^5^-1 oocysts/gram) were studied for both *C. parvum* and *C. hominis*. Ranges of dilutions were done from highly concentrated natural stool samples that we diluted subsequently. Ranges of dilutions were performed in liquid stool matrix exempt of *Cryptosporidium* species. Each sample was vortexed for 20 s before performing dilutions. Oocyst numeration was done microscopically using Kova cells and confirmed by immunofluorescence as described in Section 4.2.

Regarding the studied *Cryptosporidium* rare species, stools were selected from the CNR collection with oocyst concentrations that varied between 10^3^ and 10^4^ oocysts/gram to be easily detectable.

Other positive stool specimens were obtained from the CNR-LE collection to evaluate specificity: *Giardia intestinalis* (n = 3), *Blastocystis hominis* (n = 3), *Enterobius vermicularis* (n = 3), *Chilomastix mesnilii* (n = 3), *Entamoeba histolytica* (n = 3), *Entamoeba dispar* (n = 3), *Encephalitozoon intestinalis* (n = 3), and *Enterocytozoon bieneusi* (n = 3) positive stool samples were tested. Exact stool concentrations of these other pathogens were not calculated. Positivity was objectified by microscopy exclusively assuming relatively high concentrations. Additional tests were performed on *Candida albicans* (n = 3) and on negative stool samples (n = 20). A total of 784 PCRs were performed (98 per tested method).

### 4.2. Detection Limit Assays

Serial ten-fold oocyst dilutions (10^5^-1 oocysts/gram of stool) were performed using a negative liquid stool human matrix. Each corresponding dilution was confirmed by counting oocysts microscopically both on Kova slides (Labellians, Nemours, France) and using Crypto-Cel FITC (Cellabs, Sydney, Australia) staining according to the manufacturer’s instructions. Limits of detection were estimated considering the positivity of at least one of the three tested replicates per condition. *Cryptosporidium* negativity of the matrix was based on both microscopy and PCR investigations from the method described by Valeix et al. 2020.

### 4.3. DNA Extraction

Based on a previous published work [22], we chose an extraction protocol offering highly satisfying performances in *C. parvum* DNA extraction from stool matrix. Accordingly, the observed PCR performances were exclusively due to amplification methods since extraction was standardized. Consequently, DNA extraction was performed using a QIAamp PowerFecal DNA kit (Qiagen, Courtaboeuf, France) according to the manufacturer’s instructions. Briefly, it is a manual extraction kit combining thermal, mechanical, and chemical lysis. The starting volume for DNA extraction was 250 µL of sample. Obtained DNA extracts (100 µL) were stored at −20 °C until use. In addition, to control DNA extraction, we used Diacontrol DNA^®^ for each sample according to the manufacturer’s instructions. Ten microliters of viral DNA control was inoculated in each sample before extraction. Control DNA was subsequently detected using ready-to-use ProbePrimer mix (DICD-CY-L100) with the following PCR protocol: 50 °C for 2 min; 95 °C for 10 min; 95 °C for 15 s and 60 °C for 60 s, repeated 45 times.

### 4.4. PCR Testing

Eight real-time PCR methods were tested on the same DNA extracts: 4 “in-house” PCRs already assessed [13,14,15,22] and 4 multiplex commercial PCRs: RIDA^®^ GENE Parasitic Stool Panel II (R-Biopharm, Darmstadt, Germany), FTD^®^ Stool parasites (Fast Track Diagnostics, Esch-sur-Alzette, Luxembourg), Amplidiag^®^ Stool Parasites (Mobidiag, Paris, France), and Allplex^®^ GI Parasite Assay (Seegene, Düsseldorf, Germany). The studied methods were selected based on methods used in France according to data collected by the CNR-LE for cryptosporidiosis. PCR was performed in triplicate for each tested condition on a CFX96 PCR detection system (Bio-rad, Marnes-la-Coquette, France) according to published data for “in-house” PCR and according to the manufacturer’s instructions for commercial PCR (synthesized in Table 4). A total of 784 PCRs were performed (98 per tested method). In detail, each studied condition was extracted 3 times (N = 3) and run in simplicate per extract for PCR amplification. Consequently, regarding assays from range of dilution + rare species + specificity investigations respectively: 36 + 15 + 47 = 98 DNA extracts were tested for each studied method (N = 8). Assays were divided into two runs per studied method (two distinct PCR plates). A total of 16 PCR runs were done.

Results were considered positive when curves were exponential in logarithmic scale until the last cycle expected by each PCR program (Table 4).

PCR efficiencies (10^_1/slope^ _1) were estimated according to Bustin et al. 2009: plotting the logarithm of the initial template concentration on the x-axis and Cq on the y-axis [35]. R² values were obtained using graphical representation on Excel software.

## 5. Conclusions

Recent epidemiology confirms that cryptosporidiosis is common worldwide in both immunocompetent and immunocompromised individuals. Diagnostic approaches are still mainly based on microscopy; however, PCR-based methods are increasingly used for routine diagnosis. The performance of PCR methods is variable and needs to be evaluated. In this study, based on PCR analysis of the same DNA extracts, we compared the performance of eight commonly used methods according to limit of detection (for both *C. hominis* and *C. parvum*), specificity, and rare species identification. All eight methods were able to detect *C. parvum* and *C. hominis* with a limit of detection of 1000 oocysts/gram of stool, but only one method (FTD^®^) was able to detect one and ten oocysts/gram for *C. parvum* and *C. hominis*, respectively. Specificity was satisfactory for each tested method. Six of the eight methods were able to detect rare species implicated in human infection.

## Figures and Tables

**Table 1:** Limit of detection of tested “in-house” methods on *C. parvum* and *C. hominis*. PCR efficiencies were calculated based on obtained results from corresponding ranges of dilutions.

	Mean Cq Value (+/− Standard Deviation)					
	Oocysts/Gram	Fontaine et al. 2002	Valeix et al. 2020	Hadfield et al. 2011	Mary et al. 2013	*p*-Value
*C. parvum*	10^5^	28.47 (+/− 0.54)	27.60 (+/− 0.27)	28.14 (+/− 0.42)	24.80 (+/− 1.41)	0.15
	10^4^	31.78 (+/− 0.17)	29.86 (+/− 0.49)	31.02 (+/− 0.32)	26.97 (+/− 1.32)	0.002
	10^3^	34.71 (+/− 1.37)	35.96 (+/− 0.31)	35.72 (+/− 0.27)	30.19 (+/− 0.27)	<0.001
	10^2^	/	36.24 (+/− 0.25)	39.15	32.38 (+/− 1.8)	/
	10	/	36.64 (+/− 0.28)	/	/	/
	1	/	/	/	/	/
	Corresponding *C. parvum* PCR efficiency (%)	109	111	84.9	143	/
	R² value	0.998	0.878	0.994	0.994	/
*C. hominis*	10^5^	28.14 (0.20)	28.04 (+/− 0.25)	28.76 (+/− 0.01)	27.15 (+/− 0.04)	<0.001
	10^4^	30.9 (+/−0.25)	29.66 (+/− 0.47)	31.33 (+/− 0.09)	29.69 (+/− 0.06)	0.001
	10^3^	38.27	36.14 (+/− 0.48)	/	36.66 (+/− 0.61)	/
	10^2^	/	/	/	/	/
	10	/	/	/	/	/
	1	/	/	/	/	/
	Corresponding *C. hominis* PCR efficiency (%)	57.5	76.5	/	62.3	/
	R² value	0.939	0.893	/	0.932	/

**Table 2:** Limit of detection of tested commercial methods on *C. parvum* and *C. hominis.* PCR efficiencies were calculated based on obtained results from corresponding ranges of dilutions.

	Mean Cq Value (+/− Standard Deviation)					
	Oocysts/Gram	RIDA^®^ GENE Parasitic Stool Panel II	FTD^®^ Stool Parasites	Amplidiag^®^ Stool Parasites	Allplex^®^ GI Parasite Assay	*p*-Value
*C. parvum*	10^5^	27.61 (+/− 0.15)	19.84 (+/− 0.25)	28.02 (+/− 0.11)	28.32 (+/− 0.12)	<0.001
	10^4^	30.62 (+/− 0.25)	22.88 (+/− 0.22)	32.24 (+/− 0.15)	31.97 (+/− 0.21)	<0.001
	10^3^	37.7	26.59 (+/− 0.24)	35.17 (+/− 0.95)	34.72 (+/− 0.42)	/
	10^2^	/	30.50 (+/− 0.57)	44.2	37.71 (+/− 0.66)	/
	10	/	34.47 (+/− 1.77)	/	37.68	/
	1	/	34.61 (+/− 1.82)	/	/	/
	Corresponding *C. parvum* PCR efficiency (%)	57.8	104	56.4	156	/
	R² value	0.949	0.970	0.939	0.932	/
*C. hominis*	10^5^	27.73 (+/− 0.05)	21.41 (+/− 0.09)	29.01 (+/− 0.16)	27.39 (+/− 0.45)	<0.001
	10^4^	29.63 (+/− 0.10)	22.95 (+/− 0.09)	32.06 (+/− 0.38)	29.60 (+/− 0.09)	<0.001
	10^3^	38.53 (+/− 2.74)	26.84 +(/− 0.31)	36.49 (+/− 1.10)	33.35 +(/− 0.06)	0.003
	10^2^	/	29.22 (+/− 0.04)	44.28	36.78 (+/− 0.62)	/
	10	/	31.12 (+/− 0.28)	/	/	/
	1	/	/	/	/	/
	Corresponding *C. hominis* PCR efficiency (%)	53.1	145	58.1	105	/
	R² value	0.877	0.982	0.956	0.985	/

**Table 3:** Detection of rare species of *Cryptosporidium* implicated in human cases by tested methods.

	*C. cuniculus*	*C. meleagridis*	*C. felis*	*C. chipmunk*	*C. ubiquitum*
Fontaine et al. 2002	Yes	Yes	No	No	No
Valeix et al. 2020	Yes	Yes	Yes	Yes	Yes
Hadfield et al. 2011	Yes	Yes	Yes	Yes	Yes
Mary et al. 2013	No	No	No	No	No
RIDA^®^ GENE Parasitic Stool Panel II	Yes	Yes	Yes	Yes	Yes
FTD^®^ Stool parasites	Yes	Yes	Yes	Yes	Yes
Amplidiag^®^ Stool Parasites	Yes	Yes	Yes	Yes	Yes
Allplex^®^ GI Parasite Assay	Yes	Yes	Yes	Yes	Yes

**Table 4:** Description of tested methods.

Designation	Primers (5′-3′)	Probe (5′-3′)	Target	Amplicon Size (bp)	Thermocycling Conditions	Total Duration
Fontaine et al. 2002 [13] method	F:CGCTTCTCTAGCCTTTCATGA R: CTTCACGTGTGTTTGCCAAT	CCAATCACAGAATCATCAGAATCGACTGGTATC	Specific *C. parvum* sequence	138	50 °C—2 min 95 °C—10 min 40 cycles: 95 °C—15 s/60 °C—1 min	62 min
Valeix et al. 2020 [22] method	F: GTTAAACTGCRAATGGCT R: CGTCATTGCCACGGTA	CCGTCTAAAGCTGATAGGTCAGAAACTTGAATG and GTCACATTAATTGTGATCCGTAAAG	18S rRNA	258	95 °C—10 min 50 cycles: 95 °C—15 s/50 °C—15 s (touchdown from 60 °C)/72 °C—15 s	48 min
Hadfield et al. 2011 [14] method	F:GAGGTAGTGACAAGAAATAACAATACAGG R:CTGCTTTAAGCACTCTAATTTTCTCAAAG	TACGAGCTTTTTAACTGCAACAA	18S SSU rRNA	300	95 °C—10 min 55 cycles: 95 °C—15 s/60 °C—60 s	78 min
Mary et al. 2013 [15] method	F: CATGGATAACCGTGGTAAT R: TACCCTACCGTCTAAAGCTG	CTAGAGCTAATACATGCGAAAAAA	18S rRNA	178	94 °C—10 min 45 cycles: 94 °C—10 s/54 °C—30 s/72 °C—10 s	48 min
RIDA® GENE Parasitic Stool Panel II	Not disclosed	Not disclosed	Not disclosed	Not disclosed	95 °C—1 min 45 cycles: 95 °C—15 s/60 °C—30 s	35 min
FTD® Stool parasites	Not disclosed	Not disclosed	DNA J-like protein gene	Not disclosed	50 °C—15 min 94 °C—1 min 40 cycles: 94 °C—8 s/60 °C—1 min	62 min
Amplidiag® Stool Parasites	Not disclosed	Not disclosed	COWP gene	Not disclosed	95 °C—10 min 45 cycles: 95 °C—15 s/65 °C—1 min	66 min
Allplex® GI Parasite Assay	Not disclosed	Not disclosed	Not disclosed	Not disclosed	50 °C—20 min 95 °C—15 min 45 cycles: 95 °C—10 s/60 °C—1 min/72 °C—30 s	110 min

F: Forward. R: Reverse. SSU: Small subunit. COWP: *Cryptosporidium* oocyst wall protein.

## Data Availability

Not applicable.
